# Country over-citation ratios

**DOI:** 10.1007/s11192-017-2490-z

**Published:** 2017-08-21

**Authors:** Victoria Bakare, Grant Lewison

**Affiliations:** Department of Cancer Studies, Guy’s Hospital, King’s College London, Great Maze Pond, London, SE1 9RT UK

**Keywords:** Citing papers, Country data, Evaluation, Self-citations

## Abstract

There is a clear tendency for authors of scientific papers to over-cite the papers by their fellow countrymen (and countrywomen) relative to the percentage presence of their papers in world output in the same field. We investigated the Over-Citation Ratio (OCR) as a function of this percentage, and the effects of different scientific fields and publication years. For cancer research, we also compared clinical with basic research. We found that the OCR for a given percentage presence has been decreasing over the period 1980–2010, probably because of better communications. It is greater for fields of relatively more national interest (chemistry, ornithology) and less for those of international concern (astronomy, diabetes, cancer). It may also be slightly greater for basic cancer research than for clinical work. The OCR values given allow other types of citation, such as the references on clinical practice guidelines and papers featured in newspaper stories, to be put in context: are they unusually nationalistic, or typical of normal citation behaviour?

## Introduction

This paper is concerned with the practice whereby researchers from a given country tend to over-cite research by their fellow-countrymen (and, of course, their fellow-countrywomen). In practice, we investigate how often research by a given country is cited by papers from the same country. One might suppose that if a country’s research in a particular field of science represented (say) 1% of world output, then a “fair” quota of citations from the country would be 1% of the total. In practice it is almost always more than this, so we say that the percentage of the citing papers, divided by the country’s presence in the field, is the over-citation ratio, denoted as OCR.

Why is this important? There are two main reasons. First, the opposite side of the coin, so to speak, is the percentage of citations that come from *other* countries, which could be an indicator of the international impact of the work. This indicator needs to be carefully normalised to take account of the various factors that can influence it, and the paper goes into some detail about these. The second reason for the study is that the OCR for research papers cited by papers in the serial literature provides a benchmark for the evaluation of other types of citations. For example, Grant et al. (2000) noted that a sample of 15 UK clinical practice guidelines (CPGs) had over-cited British research papers that contributed to their evidence base by a factor of 2.5. Was this unusually high or unusually low? The authors didn’t say, because there was no benchmark to guide them. Similarly, Lewison et al. (2008) found that the British Broadcasting Corporation’s website over-cited UK cancer research papers by a factor of six, but the time period involved and the subject matter were different from those of the papers found by Grant et al.

In this paper we proceed to investigate the country OCR as a parameter of different sets of papers: how it depends on the country of authorship of the papers, their date, and their subject matter and research level (from applied or clinical to basic research). It is not meant to provide exhaustive data but rather to describe a methodology that can be applied to a given set of papers. It will also give enough results that the main features of OCR can be discerned, and used to show if any given value is higher or lower than expected. Two hypotheses will be explored: the first, the subjects of relatively local or national interest will lead to higher country OCRs than ones of international interest, and second, that in biomedical subjects, clinical papers will also give higher OCRs than ones on basic research.

Self-citation can take place at many different levels. The one most commonly addressed in the literature is that of the individual author, who may be thought to boost her ranking by this means. But it appears that author self-citations may be correlated with higher citations by others (Fowler and Aksnes 2007), and they do not account for the increase in the citation of multi-national papers (van Raan 1998). Although some authors argue that they should be discounted (Ferrara and Romero 2013) there is little evidence that their removal would affect most indicators (Glänzel et al. 2006). Self-citation can also occur at the institutional level (Thijs and Glänzel 2006; Hendrix 2009; Gul et al. 2017) and at the language level, especially in the social sciences (Yitzhaki 1998; Egghe et al. 1999). It has been suggested that journals can improve their impact factors through the encouragement of journal self-citation or the publication of editorials and letters, whose citations count in the numerator but whose numbers do not count in the denominator of the impact factor calculation. For a few this may happen (Nisonger 2000; Campanario and Gonzalez 2006), but the practice does not appear to be widespread (Andrade et al. 2009) or that journal self-citation rates correlate negatively with overall impact factors (Motamed et al. 2002). At the highest level of self-citation, namely country OCR, there have been very few papers so far (Minasny et al. 2010; Jaffe 2011). The latter paper noted a recent increase in country self-citation rates but, as we will show, there may be a good reason for this and it does not necessarily betoken a decline in international scientific impact.

## Methodology

The analysis is based on data from the Web of Science (WoS)^©^ Clarivate Analytics for six scientific fields: astronomy, birds, cancer, chemistry, diabetes and engineering. The sets of papers included just articles, and were taken from seven publication years: 1980, 1985, 1990, 1995, 2000, 2005 and 2010. For each combination of field and year, papers from the top 20 countries (in 2010) were considered. For each cohort, the citation analysis was provided by the WoS, and the collection of citing papers was then examined. Those from the five years following the publication of the cited papers, including the publication year, were isolated, and the numbers from the given country were counted as a percentage of this total number. This percentage was compared with the country’s percentage presence in the field in the given year to give the over-citation ratio, all the counts being integer country counts of papers. However, only countries with 1% or more of the world total of papers in the given year and with an OCR <100 were retained for our analysis as there would have been too much scatter for countries with smaller outputs.

The fields of astronomy and birds were defined by means of filters containing a simple list of specialist journals and another list of title words. Examples of title words for these two fields are given below.

$$ {\text{Astronomy}}:{\text{ TI}} = \left( {{\text{ASTEROID or BOOTES or COMPACT-BINARY\,or DARK-MATTER}}} \right) $$

$$ {\text{Birds}}:{\text{ TI}} = \left( {\text{AVIAN or BIRDSONG or CORMORANT or DUCKS or EAGLE}} \right) $$

These two fields were chosen to be as international as possible (astronomy), and relatively local or regional (birds). The filters for cancer and diabetes papers were similarly defined but with much longer lists: that for cancer had 185 specialist oncology journals and 323 title words or phrases. On the other hand, the filters for chemistry and engineering were based on the topic function in the WoS: TS = CHEMISTRY and TS = ENGINEERING. In cancer, a distinction was made between clinical papers and basic ones, based on words in their titles (Lewison and Paraje 2004) so that we could see if country OCR differed between three groups: papers classed as clinical (but excluding basic ones), papers classed as basic (but excluding clinical ones), and papers classed as both clinical and basic. So there were effectively eight different subject areas used in this study.

The OCR for each of the 20 countries was plotted against the country’s presence in the field in the given year (*n*, %). These graphs were on log–log scales, and a best-fit regression line was added with a power law: OCR = M × n^J.^ For all these graphs, the correlation was usually very good, typically with r^2^ ~ 0.9. The multiplier M in the equation giving the best fit was then plotted against time: this represented the OCR for a country publishing 1% of world papers. From the best-fit equation we also calculated the estimated value of OCR for a country publishing 3 and 10% of world papers.

## Results

**Fig. 1:**
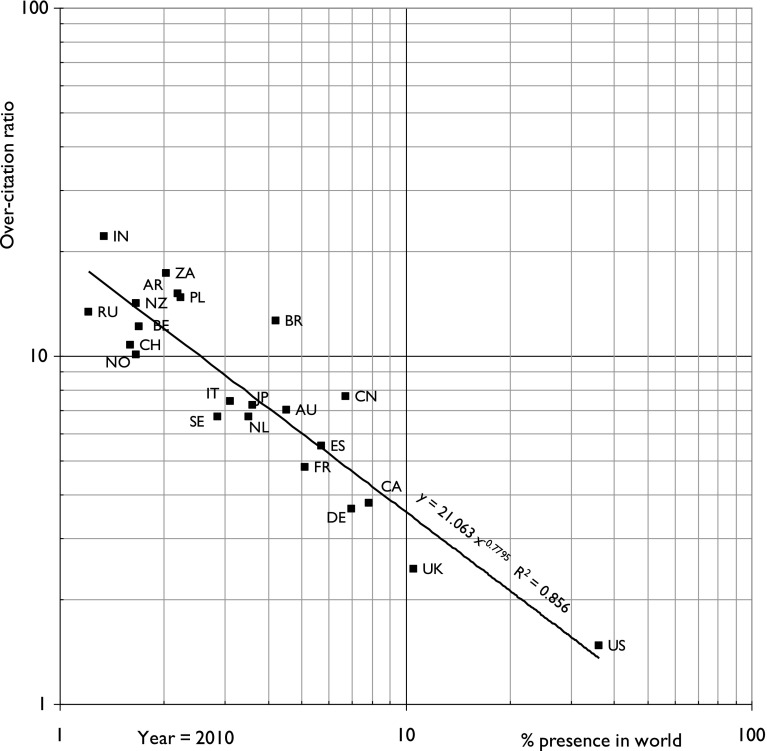
Plot of country over-citation ratio of its own papers in ornithology (research on birds) for 2010 for the 20 leading countries as a function of the country’s percentage presence in world ornithology research in that year. For country ISO2 codes, see Table 1

We were initially somewhat surprised that the correlation between a country’s percentage presence in research in a given subject area and its OCR was so strong; for years in which several of the top 20 countries were publishing <1% of the total, the correlation was even stronger when these were omitted from the analysis. Figure 1 shows a fairly typical result, that for research on birds in 2010.

The least-squares correlation line was given by a power law relationship of the type:

$$ {\text{OCR }} = {\text{ M }} \times {\text{ p}}^{J} $$

where M is a multiplier, and equal to the OCR for *p* = 1%, p is the country’s percentage presence and J is an index. It rapidly became apparent that although the index, J, varied somewhat from year to year, it tended to become less negative with time. However, M, the multiplier, was substantially greater for the earlier years than for the later ones, clearly indicating that country OCR was tending to become less pronounced in later years. This is probably because improved communication means that the diffusion of knowledge has become more international recently. These trends were found for all five of the different scientific fields (ASTRO, BIRDS, CHEM, DIABE, ENGR).

**Fig. 2:**
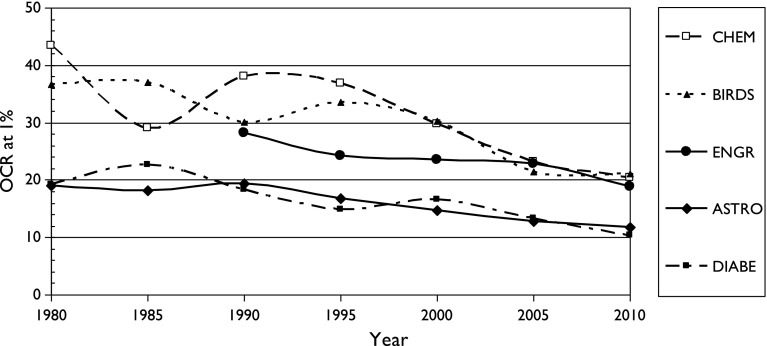
Variation of Over-Citation Ratio (OCR) with time for a country publishing 1% of world research in five scientific fields

**Fig. 3:**
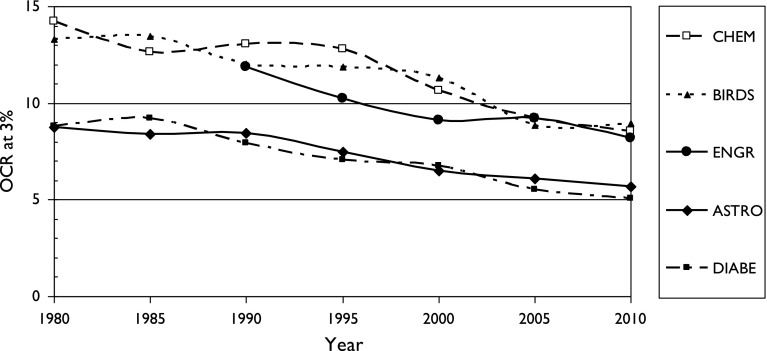
Variation of Over-Citation Ratio (OCR) with time for a country publishing 3% of world research in five scientific fields

**Fig. 4:**
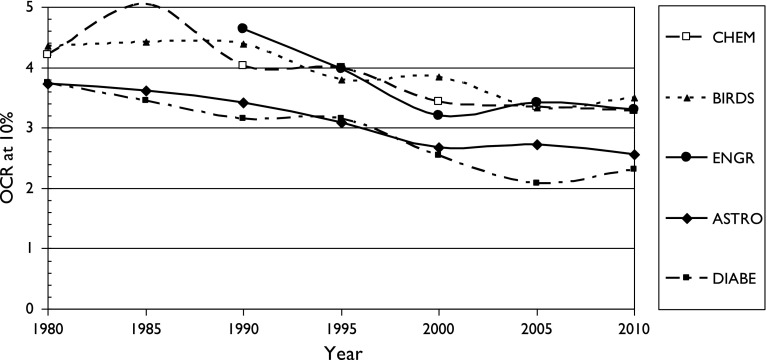
Variation of Over-Citation Ratio (OCR) with time for a country publishing 10% of world research in five scientific fields

Figures 2, 3 and 4 show the variation of M, the OCR for a country with 1, 3 and 10% of world output, respectively, with time for the five fields of research. (Note the scale change of the ordinate axis between the three graphs.) This decline is very evident, although there is some fluctuation from year to year.

**Fig. 5:**
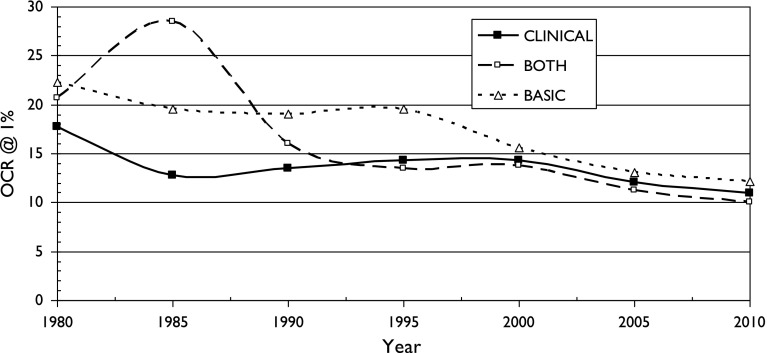
Variation of Over-Citation Ratio (OCR) with time for a country publishing 1% of world research in three research domains of oncology

**Fig. 6:**
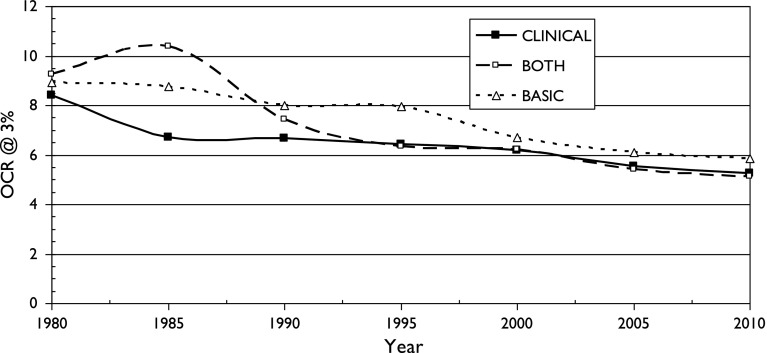
Variation of Over-Citation Ratio (OCR) with time for a country publishing 3% of world research in three research domains of oncology

**Fig. 7:**
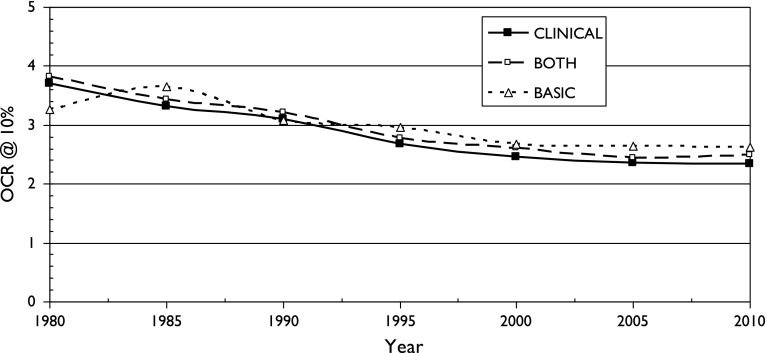
Variation of Over-Citation Ratio (OCR) with time for a country publishing 10% of world research in three research domains of oncology

There is also a fairly consistent pattern: OCR is highest for two fields with particularly local interests (chemistry, birds) and lowest for two with a more international outlook (astronomy, diabetes). It also appears for the three sets of cancer research papers, see Figs. 5, 6 and 7. It is also shown in terms of the mean values of M (OCR at 1%) and J, the index, shown in Table 2.

**Table 1:** List of leading countries whose research outputs were analysed for this study, with their ISO2 digraph codes

Country	ISO	Country	ISO	Country	ISO
Argentina	AR	Spain	ES	Poland	PL
Australia	AU	France	FR	Portugal	PT
Belgium	BE	Hungary	HU	Russia	RU
Brazil	BR	India	IN	Sweden	SE
Canada	CA	Italy	IT	Singapore	SG
Switzerland	CH	Japan	JP	United Kingdom	UK
Chile	CL	Mexico	MX	United States	US
China (P.R.)	CN	Netherlands	NL	South Africa	ZA
Germany	DE	Norway	NO		
Denmark	DK	New Zealand	NZ

**Table 2:** Mean values of M (the multiplier, or OCR for 1% presence) for 1990–2010, and of J (the index) for five major fields and three sets of cancer research (ONCOL)

Subject	M (90–10)	J (index)	Subject	M (90–10)	J (index)
CHEM	29.7	−0.90	ONCOL clinical	13.0	−0.70
BIRDS	27.3	−0.87	ONCOL both	12.9	−0.68
ENGR	23.5	−0.80	ONCOL basic	15.9	−0.75
ASTRO	15.1	−0.71			
DIABE	14.7	−0.75			

These three graphs show that there is surprisingly little difference in the over-citation ratio for the three groups of cancer research papers. In the 21st century, for which there are more countries, and many more papers, the pattern suggests that basic research is over-cited by own countrymen slightly more than clinical work, but the differences are much smaller than for the different scientific fields discussed previously. The mean values of M and J shown in Table 2 indicate that cancer research is rather similar in terms of OCR to diabetes research, another medical specialty, and that there is no obvious correlation with the research level of the papers.

## Discussion

Even with the large numbers of papers in the WoS in the various subject areas that we have considered, there is rather a lot of scatter in the results. This is seen in the figures where the lines do not form clear patterns either with time or with subject area. Nevertheless, there are three very clear conclusions:

- OCR is much greater for countries with small scientific output. There is a negative power relationship of the form, *OCR* = *M p* ^*J*^ where *p* is the percentage presence of the country in the field, *M* is a multiplier between 10 and 30, and *J* is an index between −0.68 and −0.90;
- OCR has tended to decrease with time between the 1980s and 2005–2010, probably because of easier (and cheaper) international communications;
- OCR is higher for scientific fields of more national interest, such as ornithology (and chemistry) and lower for ones that are more universal, such as astronomy and medicine.

The paper describes a methodology that can be applied to any selected field and it gives results that show the extent of own country OCR in scientific papers in the serial literature. These values will provide a baseline with which the over-citation ratio of other document types can be compared to show whether they are more, or less, nationalistic than the countries’ researchers.
